# Characterizing median nerve deformation and recovery processes in response to repeated isometric wrist torque exertions

**DOI:** 10.7717/peerj.21724

**Published:** 2026-09-14

**Authors:** Shengwei Li, Aaron M. Kociolek, Ping Yeap Loh

**Affiliations:** 1 Graduate School of Design, Kyushu University, Fukuoka, Japan; 2 School of Physical and Health Education, Nipissing University, North Bay, Canada; 3 Department of Human Life Design and Science, Faculty of Design, Kyushu University, Fukuoka, Japan

**Keywords:** Median nerve, Carpal tunnel, Wrist torque, Ultrasonography, Post-load recovery

## Abstract

**Background:**

This study examined median nerve deformation during isometric wrist torque and characterized time-dependent recovery following load release.

**Methods:**

Twenty-five healthy adults performed isometric wrist torque exertions at 50% maximal voluntary torque in four torque-grip experimental conditions (flexion with gripping, FG; extension with gripping, EG; flexion without gripping, FN; and extension without gripping, EN). Each torque-grip condition was repeated in 10 consecutive trials. For each trial, ultrasound images were acquired prior to torque production (baseline), at 50% maximal voluntary torque maintenance, and 0.25, 0.5, 1.0, and 2.0 s after torque release. Median nerve cross-sectional area (MNCSA) was measured to quantify deformation and recovery processes as a function of torque direction, gripping, time, and repetition.

**Results:**

For all torque-grip conditions, MNCSA decreased during torque maintenance compared to baseline (*p* < 0.001), indicating acute cross-sectional deformation consistent with compression. No significant differences were found between flexion and extension, or between gripping and non-gripping, during torque maintenance. After torque release, MNCSA recovered progressively but remained below baseline at 2.0 s. Repetition did not alter the overall recovery pattern, though a small difference appeared at 1.0 s in the EN condition between initial and post-repeated trials. A post-repetitive difference between FN and EN was also observed at 1.0 s.

**Conclusion:**

Isometric wrist torque produces an immediate reduction in MNCSA, followed by a gradual rather than instantaneous recovery. While the effect of repeated gripping was small, minor deviations after repetition may indicate early cumulative mechanical loading. Dynamic and recovery-based assessments may provide additional insight for detecting early nerve compression risk.

## Introduction

The median nerve within the carpal tunnel has attracted increasing attention in recent years because its morphological deformation is closely associated with the onset of carpal tunnel syndrome (CTS), one of the most common peripheral neuropathies (Ibrahim et al., 2012; Genova et al., 2020). The carpal tunnel is a narrow passage formed by the carpal bones and the transverse carpal ligament, through which the median nerve and several flexor tendons pass (Presazzi et al., 2011). Ultrasound assessment of the median nerve has been widely applied in both clinical diagnosis and experimental research, and previous studies have demonstrated its usefulness for detecting nerve swelling and morphological changes under mechanical loading (Yoshii et al., 2009; Yao & Roll, 2022; Xu et al., 2024). However, most existing evaluations are based on static measurements of the median nerve, typically the median nerve cross-sectional area (MNCSA) used as a diagnostic threshold for CTS (Klauser et al., 2009; Impink et al., 2010; Yoshii, Zhao & Amadio, 2020). Such static criteria do not fully reflect its deformation and recovery behavior following mechanical stress and are less effective in distinguishing individuals with pathological changes in MNCSA from individuals with high but still normal values, thereby limiting the accurate assessment of the nerve’s functional condition (Ng et al., 2022; Roll et al., 2023).

To overcome the limitation of using a single static threshold, some studies have proposed relative evaluation methods based on MNCSA comparisons under different conditions. For example, the ratio of MNCSA between the proximal and distal carpal tunnel or the MNCSA difference between the carpal tunnel and the pronator quadratus region has been shown to reduce the influence of inter-individual variation through within-subject normalization, thereby providing higher diagnostic sensitivity and specificity (Klauser et al., 2009; Fu et al., 2015; Xu et al., 2022). However, although these methods partially address the limitations of a single threshold, they still overlook the effects of mechanical loading on the median nerve. In particular, previous studies have mainly examined morphological changes with varying wrist angles rather than with wrist torque, and only a few have investigated the recovery of the median nerve following mechanical loading. Previous time-varying assessments showed that changes in median nerve deformation and position during force loading were not completely reversed during force unloading (Racine, Holmes & Kociolek, 2024), while MNCSA remained below the initial baseline immediately after grip force returned to approximately 0 N (Li et al., 2025). These findings suggest that median nerve morphology continues to change after the external force has been removed and that recovery should be evaluated as a temporal trajectory across successive post-release measurements rather than inferred from a single time point. However, neither the early post-release trajectory nor the time required for complete recovery has been established. Understanding these time-dependent changes is essential, as the recovery behavior of MNCSA may provide additional information about the short-term mechanical response and resilience of the nerve beyond that obtained from static measurements.

In many occupational and daily activities, wrist torque is accompanied by grip force, with finger flexor tendons lying volar to the wrist flexion-extension axis, and their activation during gripping generating a wrist flexion torque, thereby changing mechanical loading within the carpal tunnel (Fennigkoh, Garg & Hart, 1999; Seo et al., 2008). Accordingly, the present study aimed to evaluate the effects of isometric wrist torque on median nerve deformation under both gripping and non-gripping conditions and to characterize its time-dependent recovery behavior after load release. To accomplish this objective, we quantitatively analyzed both deformation and recovery of the median nerve by constructing MNCSA recovery curves at multiple time points following isometric wrist torque. We hypothesized that torque exertion would produce a significant reduction in MNCSA, and that MNCSA would gradually recover after load release rather than return instantaneously to its baseline value.

## Materials and Methods

This study was approved by the Research Ethics Committee of the Faculty of Design, Kyushu University (approval no. 555).

### Participants

Twenty-five healthy undergraduate and graduate university students (male = 12, female = 13) were recruited *via* convenient sampling and provided written informed consent prior to their participation. Table 1 presents the participants’ demographic data. Participants were recruited and assessed as previously described (Li et al., 2025). Specifically, the only inclusion criteria were ability and willingness to provide informed consent and above 20 years of age. The exclusion criteria for participation were: (1) previous CTS, (2) wrist injury history by self-report, and (3) positive results from Phalen’s Test or Tinel’s Sign. Physical anthropometry characteristics were recorded (height and weight), and the 10-item Edinburgh Handedness Inventory was used to identify participant handedness (Oldfield, 1971).

**Table 1:** Mean (±SD) demographic data of the participants (*n* = 25).

Variable		All (*n* = 25)	Male (*n* = 12)	Female (*n* = 13)
Age (years)		26.7 ± 3.4	26.3 ± 3.3	27.2 ± 3.6
Height (cm)		169.3 ± 9.7	177.9 ± 9.7	161.3 ± 4.8
Weight (kg)		66.5 ± 18.7	73.5 ± 18.7	60.0 ± 17.5
BMI (kg/m^2^)		23.1 ± 5.8	23.1 ± 5.8	23.1 ± 6.9
Handedness	Right-handed	25	12	13

An *a priori* power analysis was conducted using G*Power software (version 3.1.9.7, Heinrich Heine University Düsseldorf, North Rhine-Westphalia, Germany), assuming an effect size of f = 0.30 and a significance level of 0.05. The required sample size was estimated to be *n* = 17 to achieve 0.80 statistical power. To enhance the study’s sensitivity, we increased the sample size to *n* = 25. This adjustment allowed the detection of smaller effects (f = 0.25) while maintaining a statistical power exceeding 0.80 or raised the statistical power to 0.94 for the same effect size.

### Experimental setup

**Figure 1:**
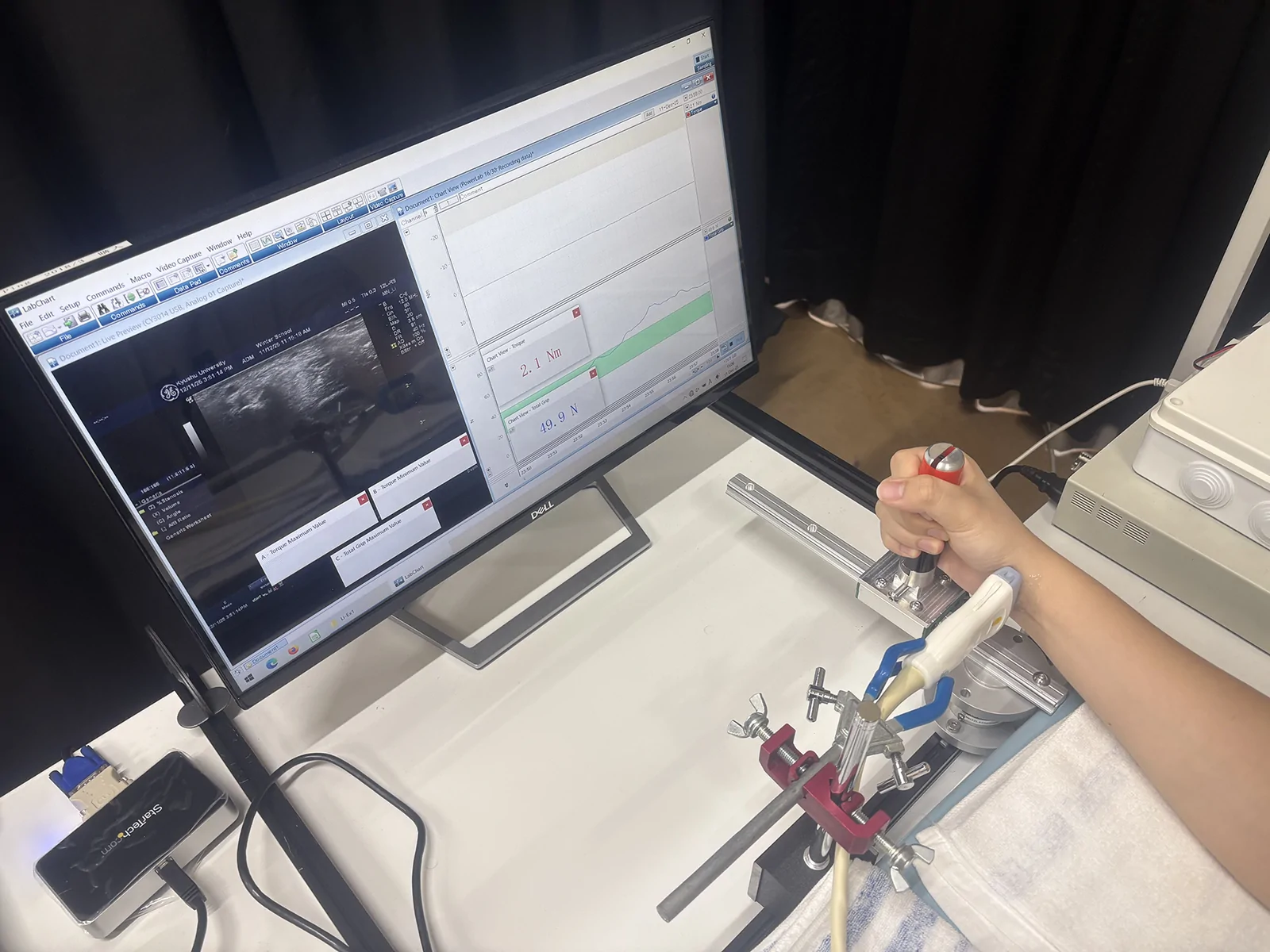
Experimental setup. Wrist torque and grip force measurement with the measurement unit. The display monitor showed real-time wrist torque of 2.1 Nm (red) and grip force of 49.9 N (blue).

A custom-built measurement unit secured to a table was used to measure wrist torque and grip force (Fig. 1). Specifically, a non-rotating torque sensor (LTMS-25; Toyo Sokki, Kanagawa, Japan) was fixed to the table to measure wrist torque, and a handle with a slider incorporating load cells (TCLS-20L; Toyo Sokki, Kanagawa, Japan) was attached to the torque sensor *via* a linear guide to measure grip force. Participants were seated facing the measurement unit with the elbow supported on a pad and gripped the handle with the wrist in a neutral position (*i.e*., 0° wrist flexion/extension and 0° radioulnar deviation). The handle position on the linear guide was adjusted and fixed so that the rotation axis of the wrist was aligned with the axis of the torque sensor (Yoshii et al., 2015; Plewa, Potvin & Dickey, 2016). Isometric wrist flexion and extension torque, as well as grip force, were measured on the dominant hand using the measurement unit, and the electrical signals were amplified (A-M8; Omega-denshi, Osaka, Japan) and sampled at 1,000 Hz using an A/D converter (PowerLab PL3508; ADInstruments, Sydney, NSW, Australia). The wrist torque and grip force were visualized and numerically displayed *via* LabChart software (version 7.3.8; ADInstruments, Sydney, NSW, Australia) on a monitor placed on the table, which provided each participant with a real-time display of the measured torque and grip force.

### Experimental procedure

Each participant completed one full experimental session, which began with a familiarization phase that included wrist positioning, torque exertion tasks, and observation of real-time wrist torque displayed on the monitor. All measurements were performed on the dominant hand. Before the main experimental session, each participant performed three maximal voluntary torque (MVT) trials for both wrist flexion and extension, and the average value of the three trials was defined as 100% MVT (3.4 ± 1.9 Nm for flexion and 2.5 ± 1.4 Nm for extension). The experimental session included ultrasound examinations at the baseline condition with light handle gripping (≈0% MVT, ≈0 N grip force) and during isometric torque exertion at a target level of 50% MVT. Once participants achieved 50% MVT, ultrasound images were sampled at five time points, including during torque maintenance and post-release phases (at 0.25, 0.5, 1.0, and 2.0 s after torque release), from continuous ultrasound recordings. Based on previous evidence of residual median nerve deformation during force unloading and immediately after force release (Racine, Holmes & Kociolek, 2024; Li et al., 2025), the serial post-release time points were selected to characterize the immediate phase of MNCSA recovery with relatively dense temporal sampling during the first 2.0 s. The 2.0 s time point represented the end of the planned observation window and was not defined as the expected time required for complete recovery.

Four torque-grip conditions were examined: wrist flexion with natural gripping (FG) and extension with natural gripping (EG), where participants were allowed to exert naturally self-selected accompanying grip force without a prescribed target level, and wrist flexion (FN) and extension (EN) without grip force (≈0 N), where participants were instructed to avoid gripping. Each condition consisted of 10 torque exertion trials, with each trial involving 5 s of torque maintenance followed by 5 s of rest. The order of the conditions was randomized across participants, and a 5-min rest period was provided between conditions to minimize the effect of fatigue accumulation. Among the 10 trials in each condition, the first three were defined as initial trials, and the last three as post-repetitive trials, with ultrasound examinations performed in both (the three trials in each stage were later averaged for statistical analysis) to evaluate median nerve deformation and recovery.

### Ultrasound image acquisition and analysis

**Figure 2:**
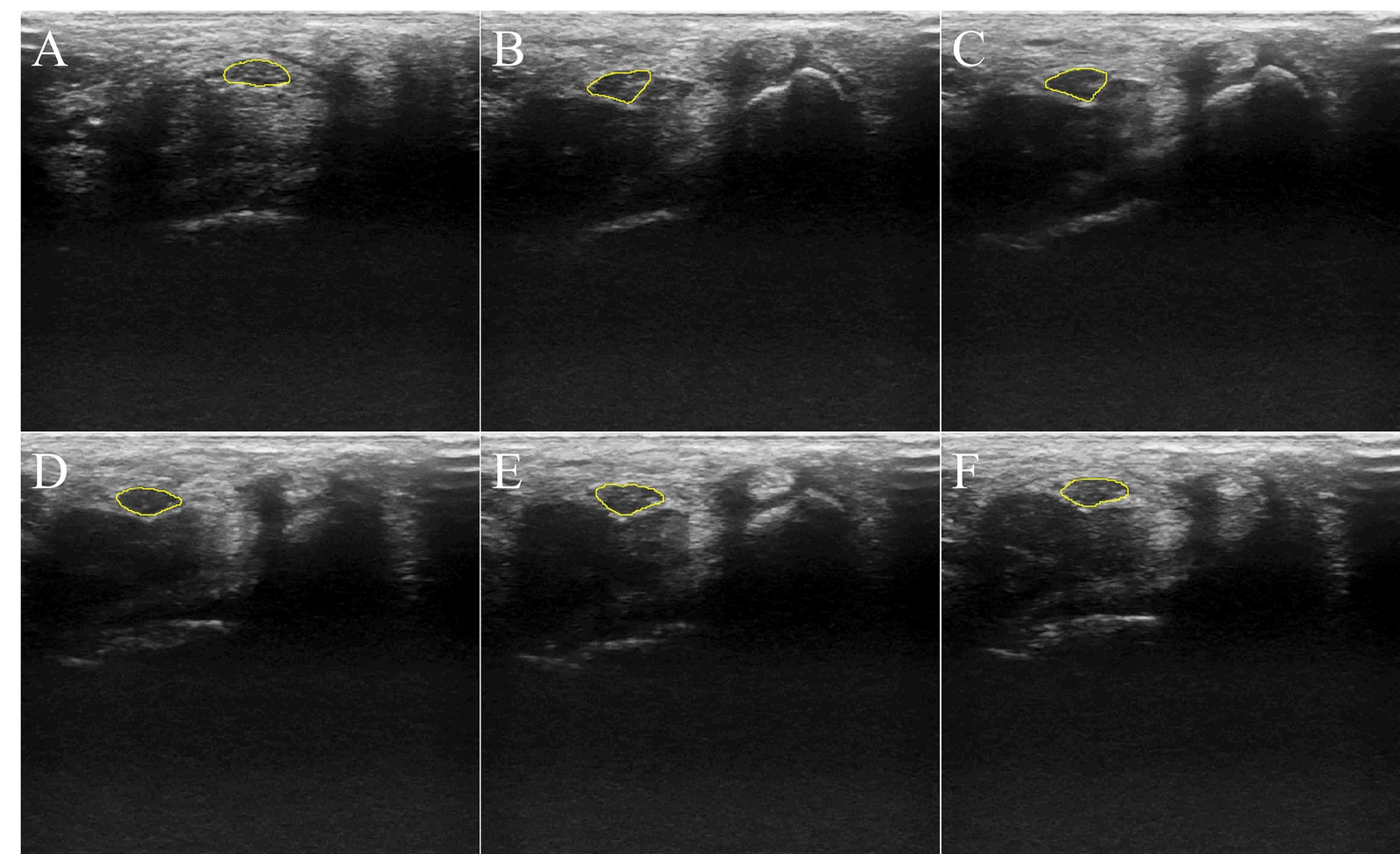
Representative ultrasound images of the median nerve under the EG condition (extension torque with grip) across the six sample points. (A) Baseline. (B) Torque maintenance. After torque release (C) 0.25 s. (D) 0.5 s. (E) 1.0 s. (F) 2.0 s. Yellow line was the outlined median nerve boundary used for MNCSA measurement.

Ultrasound imaging was conducted using a Viamo portable ultrasound system (Canon Medical Systems, Tochigi, Japan) equipped with a PLT-1204ST linear array transducer. During each measurement, a sufficient amount of ultrasound gel was applied between the transducer and the skin to ensure optimal acoustic coupling while minimizing the pressure exerted by the transducer on the underlying tissue. The probe was mounted on the measurement apparatus *via* a three-prong adjustable clamp, with its orientation fixed parallel to the distal wrist crease to visualize the median nerve in the transverse plane at the proximal carpal tunnel. The imaging parameters were set to a scanning frequency of 12 MHz and a video frame rate of 20 fps. The total scanning depth was adjusted to 30 mm, and the depth of interest (approximately 10 mm) was centered on the median nerve to enhance the visualization of its boundaries. With the synchronized acquisition of the torque sensor and ultrasound system, image samples were taken from the continuous ultrasound videos at time points during torque maintenance and at 0.25, 0.5, 1.0, and 2.0 s after torque release. Subsequently, all collected ultrasound images were analyzed by tracing the median nerve along its hyperechogenic border using ImageJ (version 1.53t; National Institutes of Health, Bethesda, MD, USA) to measure the MNCSA (Fig. 2).

### Statistical analyses

Statistical analyses were performed using SPSS software (version 24.0; IBM Corporation, Armonk, NY, USA). First, changes in MNCSA across the baseline and the four torque-grip conditions were evaluated using a one-way repeated measures ANOVA with five levels (baseline and four task conditions). A separate two-way repeated measures ANOVA (2 × 2; direction × grip) was additionally conducted to examine the factorial effects of torque direction and gripping during the torque maintenance phase.

To characterize the time-dependent behavior of MNCSA within each torque-grip condition, a one-way repeated measures ANOVA was first performed across six time points: baseline, torque maintenance, and the post-release phases (at 0.25, 0.5, 1.0, and 2.0 s after torque release), and the corresponding recovery curves were plotted. Subsequently, to evaluate the influence of repeated exertion on the recovery pattern, a two-way repeated measures ANOVA was conducted with trial stage (initial *vs*. post-repetitive, defined as the mean of the first three trials and the mean of the last three trials, respectively) and time (torque maintenance and the post-release phases) as within-subject factors. The Greenhouse-Geisser adjustment was applied when the assumption of sphericity was violated according to Mauchly’s test. Bonferroni pair-wise comparisons were used for *post-hoc* testing and statistical significance was defined at *p* < 0.05. All data are presented as the mean ± standard deviation (SD).

## Results

### Median nerve changes during torque maintenance

**Figure 3:**
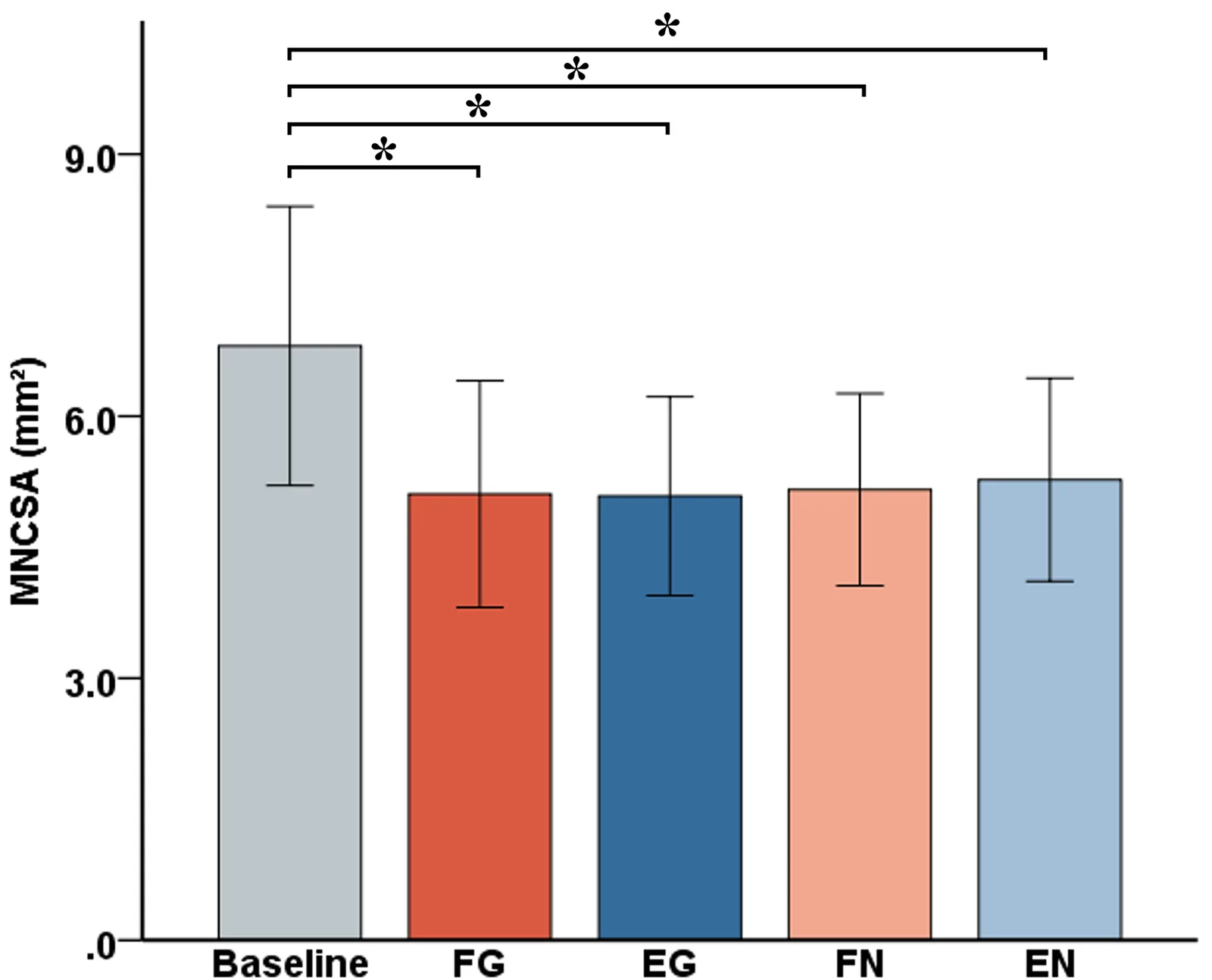
Comparison of MNCSA among baseline and the four torque-grip conditions. FG = flexion with grip, EG = extension with grip, FN = flexion without grip, EN = extension without grip, *, significant difference (*p* < 0.05).

Figure 3 shows MNCSA values under each task condition. At baseline, the mean MNCSA was 6.80 ± 1.60 mm^2^, and significantly larger than all four torque-grip conditions (all *p* < 0.001). For torque-grip conditions, the mean MNCSA were 5.11 ± 1.30 mm^2^ for FG, 5.08 ± 1.14 mm^2^ for EG, 5.16 ± 1.10 mm^2^ for FN, and 5.27 ± 1.16 mm^2^ for EN. No significant differences were found among the four conditions. The analysis showed no significant main effect of torque direction (F (1.0, 24.0) = 0.254, *p* = 0.619), and no significant main effect of grip (F (1.0, 24.0) = 1.350, *p* = 0.257). Similarly, the interaction between direction and grip was not significant (F (1.0, 24.0) = 0.528, *p* = 0.474). Specifically, MNCSA did not differ significantly between flexion and extension conditions, nor between trials performed with and without grip force.

### Median nerve recovery following torque exertion

**Figure 4:**
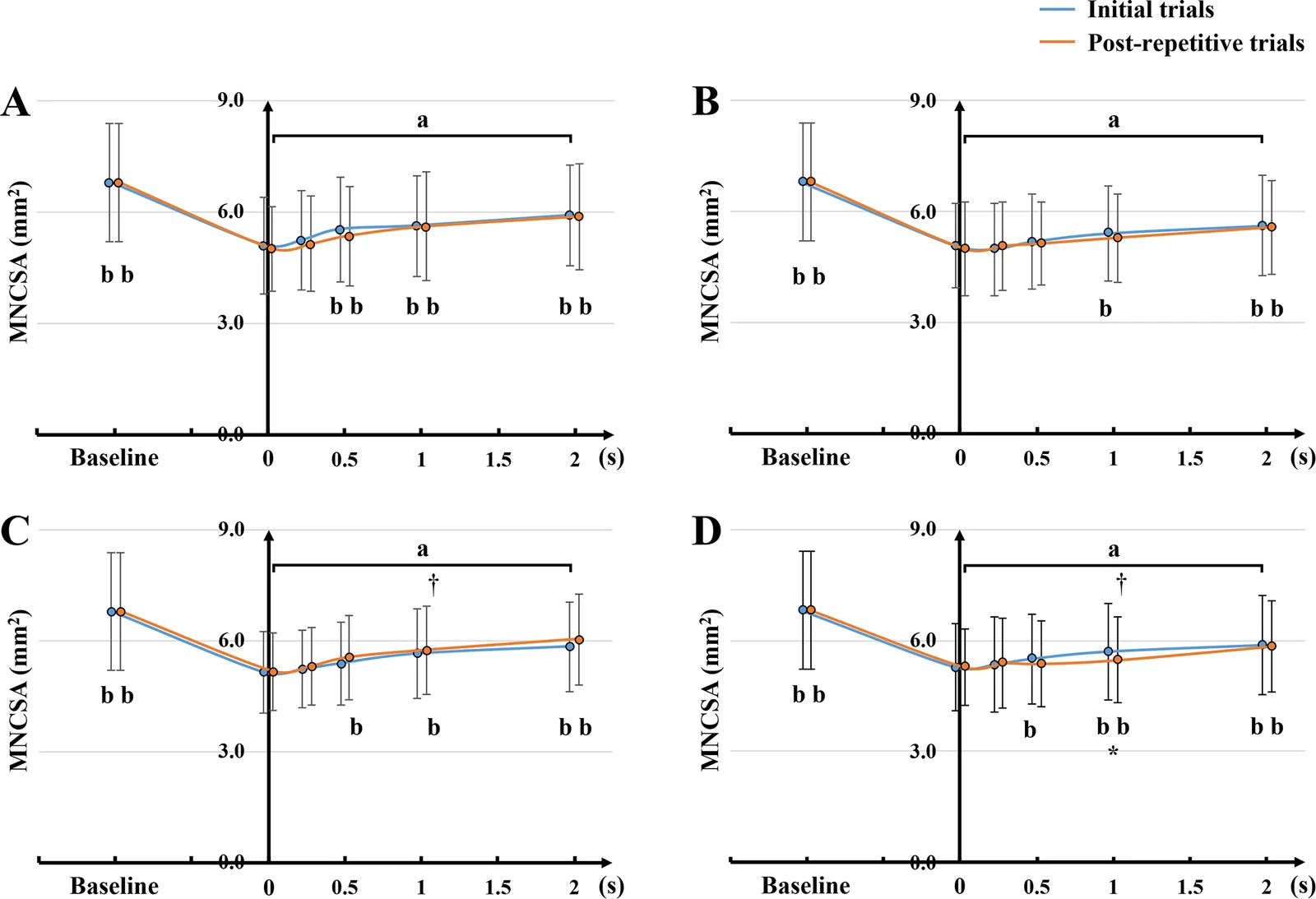
Plotted MNCSA recovery curves across torque-grip conditions. For clarity, data points from the two trial stages are slightly offset horizontally but correspond to the same actual time points. (A) FG = flexion with grip condition. (B) EG = extension with grip condition. (C) FN = flexion without grip condition. (D) EN = extension without grip condition. a, significant differences compared with baseline (*p* < 0.05), b, significant differences compared with torque release (0 s) (*p* < 0.05), *, significant difference between initial and post-repetitive trials (*p* < 0.05), †, significant difference between flexion without grip (FN) and extension without grip (EN) conditions (*p* < 0.05).

Across all torque-grip conditions (FG, EG, FN, EN) and both trial stages (initial and post-repetitive), one-way repeated measures ANOVA demonstrated a significant main effect of time in every condition (Fig. 4). For initial trials, the time effects were significant for FG, F (2.0, 48.4) = 33.965, *p* < 0.001; EG, F (2.1, 50.7) = 53.672, *p* < 0.001; FN, F (1.9, 46.6) = 35.911, *p* < 0.001; and EN, F (2.1, 49.9) = 42.174, *p* < 0.001. For post-repetitive trials, significant time effects were also observed for FG, F (2.1, 51.4) = 34.439, *p* < 0.001; EG, F (2.0, 47.0) = 49.970, *p* < 0.001; FN, F (2.0, 47.0) = 37.528, *p* < 0.001; and EN, F (1.9, 45.8) = 33.315, *p* < 0.001.

In examining whether repeated exertion influenced these responses, the main effect of trial stage was not significant for any torque-grip condition, with FG, F (1.0, 24.0) = 1.137, *p* = 0.297; EG, F (1.0, 24.0) = 0.715, *p* = 0.406; FN, F (1.0, 24.0) = 1.945, *p* = 0.176; and EN, F (1.0, 24.0) = 0.752, *p* = 0.394. Likewise, the interaction between trial stage and time did not reach significance for FG, F (3.0, 71.5) = 0.395, *p* = 0.756; EG, F (2.8, 67.1) = 0.790, *p* = 0.496; FN, F (3.0, 71.6) = 0.773, *p* = 0.512; and EN, F (2.9, 69.6) = 2.110, *p* = 0.109, indicating that repeated exertion did not produce a detectable modification in the time-dependent response pattern of MNCSA changes (Fig. 4).

In addition to these overall effects, several significant differences were observed by recovery curves. At the 1.0 s time point in the EN condition, MNCSA differed between the initial and post-repetitive trials (*p* = 0.043, Fig. 4D). At the same 1.0 s time point in the post-repetitive trials only, FN and EN also differed significantly (*p* = 0.016, Figs. 4C, 4D). Across conditions, MNCSA during the early recovery period (0.5–1.0 s) remained significantly lower than baseline, whereas MNCSA did not return to baseline by 2.0 s following torque release.

## Discussion

This study investigated the effects of isometric wrist torque on median nerve deformation and characterized its time-dependent recovery behavior after load release. The results demonstrated that isometric torque induced a clear cross-sectional deformation of the median nerve within the carpal tunnel, evidenced by a significant reduction in MNCSA during torque maintenance compared with baseline. Following torque release, the median nerve exhibited a gradual recovery rather than an instantaneous return to its baseline state, indicating a time-dependent viscoelastic response. Although no significant overall differences were found among the four torque-grip conditions, post-repetitive trials showed isolated deviations in the early recovery profile at 1.0 s, suggesting possible subtle cumulative mechanical effects on the nerve. These findings collectively confirm the initial hypothesis and highlight that the functional state of the median nerve cannot be fully evaluated by static measurements alone. The following sections discuss the mechanisms underlying median nerve compression during torque exertion and its recovery dynamics after load release.

### Effects of wrist torque on median nerve deformation

Across all torque-grip conditions, isometric wrist torque immediately reduced MNCSA, indicating acute median nerve deformation consistent with compression within the carpal tunnel (Fig. 3). This pattern is consistent with previous reports showing that wrist loading or changes in tendon tension can decrease MNCSA even in a neutral wrist posture (Turcotte & Kociolek, 2021; Li et al., 2025). The observed reduction may reflect increased tendon tension and transverse plane displacement within the tight confines of the carpal tunnel, which can transiently compress the median nerve or reduce its available cross-sectional space (Lopes et al., 2011; Van Doesburg et al., 2012; Yao & Roll, 2022). No significant differences were observed between flexion and extension torque, regardless of whether the task was performed with or without grip. Although previous work on carpal tunnel pressure has reported that wrist flexion and extension may produce different degrees of intracarpal pressure elevation, the present findings indicate that when torque magnitude is standardized and the wrist is maintained in a neutral position, both directions impose a comparable compressive effect on the median nerve (Gelberman et al., 1981; Dung Tran, 2017). This may relate to the relatively fixed bony boundaries of the carpal arch in a neutral posture, where either torque direction can change the shape and increase the pressure within the carpal tunnel through coactivation of wrist muscles and corresponding tendon loading (Yoshioka et al., 1993; Bower, Stanisz & Keir, 2006; Erhart et al., 2020). Therefore, the presence and magnitude of wrist torque, rather than direction-specific tendon paths or shape changes of the carpal tunnel, appear to be the primary determinants of acute nerve deformation.

Despite the common expectation that grip increases carpal tunnel pressure, the gripping conditions in this study did not produce significantly different MNCSA values compared with the no-grip torque conditions (Fig. 3). Earlier work involving maximal or sustained gripping has demonstrated substantial median nerve deformation, likely due to pronounced flexor-tendon bulging and elevated tunnel pressure (Loh et al., 2018; Li et al., 2025). In this study, however, the grip effort present during the torque tasks was moderate and naturally occurring, and the finger-flexor muscles were also activated to maintain wrist stability during the isometric posture, even when the task was performed in the without-grip conditions including flexion and extension (Seo et al., 2008; Popp et al., 2023). Such concurrent activation may attenuate the difference between the grip and no-grip conditions attributable to finger-flexor loading. Although a trend toward smaller MNCSA values was observed during the grip conditions, the difference did not reach statistical significance, possibly reflecting that the wrist-loading component of the task exerted a greater influence on MNCSA than the accompanying spontaneous grip force.

### Recovery behavior of the median nerve following torque release

After torque release, MNCSA increased progressively during the observed early recovery period but remained below baseline at the end of the 2.0 s observation window (Fig. 4). This indicates that after the rapid early period, the later period of recovery extended beyond the 2.0 s observation window. The observed time-dependent pattern may reflect the viscoelastic response and tissue rearrangement of the adjacent subsynovial connective tissue (SSCT) and surrounding nerve sheath following load release (Main et al., 2011; Racine, Holmes & Kociolek, 2024). Both torque directions showed substantial recovery between 0.5 and 1.0 s, after which recovery continued more slowly through the end of the 2.0 s observation window. Although gripping did not significantly alter MNCSA during torque maintenance, isolated differences emerged during recovery. At the 1.0 s time point under the post-repetitive conditions, FN exhibited significantly different MNCSA from EN, suggesting that though recovery curves were largely comparable between flexion and extension conditions, different torque directions may still induce distinct patterns of tendon displacement and subtle changes in carpal tunnel morphology, leading to a small difference in the short-term viscoelastic response (Figs. 4C, 4D).

Specifically, during wrist flexion exertion, primarily generated by the flexor carpi radialis (FCR) and flexor carpi ulnaris (FCU), the flexor tendons shift volarly, the transverse carpal ligament (TCL) arch height increases, and the distance between the TCL and the tendons decreases (Gabra et al., 2016; Ikeda et al., 2025). These changes suggest that the flexor tendons contact the TCL and displace it volarly, producing a more rounded carpal tunnel shape (Yoshioka et al., 1993). In contrast, during wrist extension exertion, driven mainly by the extensor carpi radialis brevis (ECRB) and extensor carpi ulnaris (ECU), the flexor tendons move dorsally, the carpal bones rotate dorsally, and the TCL arch is flattened, resulting in a more planar tunnel configuration (Yoshioka et al., 1993; Gabra, Domalain & Li, 2012; Yoshii et al., 2015; Gabra et al., 2016; Ikeda et al., 2025).

Peripheral nerve connective-tissue architecture may also help explain the observed recovery behavior. An *in vivo* rat median nerve stretch-to-failure model demonstrated an outside-in sequence of mechanical failure in which the epineurium failed before the endoneurial contents, whose undulating course provides reserve length during elongation (Schroen et al., 2025). Although longitudinal nerve excursion was not measured in the present study, previous ultrasound studies have demonstrated median nerve motion relative to the adjacent flexor tendons during wrist and finger activity (Lopes et al., 2011; Turcotte & Kociolek, 2021; Yao & Roll, 2022). Thus, some endoneurial reserve length may have been recruited through partial straightening of the undulating endoneurial tubes, concurrently with transverse nerve deformation reflected by the reduction in MNCSA. However, we consider near exhaustion of this reserve unlikely under the present experimental conditions. Unlike the rat stretch-injury model, the median nerve was neither mechanically fixed nor forcibly elongated to failure and remained able to glide relative to the flexor tendons and SSCT, allowing load redistribution through coordinated but nonuniform tissue motion and SSCT shear. Because the epineurium is the first connective-tissue structure to fail during severe stretching, its time-dependent viscoelastic recoil, together with that of the surrounding SSCT and endoneurial contents, may have contributed to the gradual recovery of MNCSA. Accordingly, the time-dependent recovery should not be attributed to epineurial stiffness alone, but may reflect coupled viscoelastic recoil and tissue rearrangement across these tissues. Subsequent second-harmonic generation (SHG) imaging demonstrated disorganized but continuous epineurial collagen fibers adjacent to areas of epineurial disruption following severe stretch injury (Schroen et al., 2026). Although the loading in the present study was substantially less severe, repeated subfailure deformation could conceivably produce subtle disorganization of epineurial collagen fibers that accumulates across repeated exposures. Direct measurements of endoneurial strain, epineurial mechanical properties, and collagen architecture are required to test these interpretations.

### Influence of repeated exertion and implications for repetitive loading

A significant difference appeared at the 1.0 s time point in the EN condition between the initial and post-repetitive trials (Fig. 4D). Although the overall recovery behavior was consistent across conditions, this isolated difference suggests that subtle cumulative effects may begin to manifest locally, even if the global recovery curve remains stable. This suggests that the cumulative loading imposed by the current protocol with 10 trials was sufficient to induce detectable mechanical adaptation or fatigue-related effects within the median nerve or SSCT. Prior studies examining repetitive tendon gliding or prolonged wrist loading have shown measurable increases in SSCT shear strain or delayed recovery under larger cumulative loads, longer durations, or higher repetition frequencies (Filius et al., 2017; Wong, Farias Zuniga & Keir, 2023).

In this context, the present findings may reflect the early stages of such cumulative responses, where modest repetition produces small but detectable deviations in the recovery profile before more pronounced alterations emerge. Whether recurrent deformation becomes harmful is likely to depend on its magnitude, frequency, duration, recovery interval, and individual susceptibility. If comparable loading recurred over many weeks and recovery between exposures became incomplete, repeated median nerve deformation and tendon–SSCT shear could promote fibrotic remodeling, reduce normal tissue gliding, and create a progressively less compliant mechanical environment within the carpal tunnel (Festen-Schrier & Amadio, 2018). In animal models, SSCT injury has been followed by increased fibroblast density and collagen fiber size together with reduced median nerve motor amplitude after 12 weeks, while chronic nerve compression has induced local demyelination and remyelination associated with altered nerve function (Gupta et al., 2004; Moriya et al., 2011). These findings provide a biologically plausible pathway through which recurrent mechanical loading could contribute to CTS, but they do not establish that the acute MNCSA changes observed in the present study would necessarily produce this outcome. Longitudinal studies combining longer exposure and recovery periods with assessments of symptoms, nerve conduction, perfusion, and carpal tunnel pressure are required to determine the clinical significance of this response.

### Limitation

A notable limitation of the present work is that the accompanying grip force was not experimentally controlled. The accompanying grip force recorded during flexion (60.8 ± 34.7 N) and extension (29.8 ± 19.4 N) represented 25.0% and 12.2% of mean maximal voluntary force (MVF, 243.6 ± 80.6 N), respectively. However, previous studies indicate that the primary factor influencing median nerve deformation is the presence or absence of grip force exertion, rather than the specific level of grip force (Li et al., 2025). Therefore, wrist torque appears to be the dominant contributor to acute median nerve deformation during isometric wrist torque tasks, whereas spontaneous accompanying gripping likely plays only a minor role. Second, the participants were healthy young adults, with no history of CTS and negative Phalen’s and Tinel’s tests. This relatively homogeneous sample was appropriate for characterizing the acute mechanical response of the median nerve under healthy conditions, but limits the generalizability of the findings to older adults, individuals with CTS, or populations at risk of occupational injury. The post-release observation period was limited to 2.0 s. Although the serial measurements characterized the temporal trajectory of the early recovery response, MNCSA had not returned to baseline by the end of the observation window. Consequently, the later phase of recovery and the time required for complete recovery could not be determined. Future studies should extend post-release measurements until MNCSA stabilizes or returns to baseline.

## Conclusions

This study demonstrated that isometric wrist torque reduces MNCSA, consistent with nerve compression within the carpal tunnel. Analysis of the recovery curves further revealed that the median nerve does not return immediately to its baseline state after torque release; instead, it revealed a time-dependent response characterized by an initial rapid recovery phase, after which recovery continued more slowly through the end of the 2.0 s observation window. The dynamic and repetition-related changes observed in this study may reflect early signs of median nerve compression, potentially mediated by an altered viscoelastic response of the adjacent SSCT. These findings highlight the importance of assessing not only deformation during loading but also the temporal characteristics of post-load recovery, which may provide functional insight beyond static thresholds.

## Supplemental Information

10.7717/peerj.21724/supp-1 Supplemental Information 1 Raw data for median nerve cross-sectional area (MNCSA) measurements across all participants, conditions, and time points. raw MNCSA measurements (mm^2^) for all participants, including baseline, initial trials, and post-repetitive trials across different conditions and time points. Each row corresponds to one observation used for statistical analysis.

10.7717/peerj.21724/supp-2 Supplemental Information 2 Participant demographic raw data. Individual-level demographic and anthropometric data for the 25 participants, including anonymized participant ID, gender, age, height, weight, and body mass index (BMI).

## Additional Information and Declarations

### Data Availability

The following information was supplied regarding data availability:

The data is available in the Supplemental Files.

## References

[ref-1] Bower JA, Stanisz GJ, Keir PJ (2006). An MRI evaluation of carpal tunnel dimensions in healthy wrists: implications for carpal tunnel syndrome. Clinical Biomechanics.

[ref-2] Dung Tran T (2017). Intratunnel pressure measurement in patients with carpal tunnel syndrome in Vietnam. Journal of Neurology, Neurological Science and Disorders.

[ref-3] Erhart J, Unger E, Schefzig P, Varga P, Hagmann M, Ristl R, Hajdu S, Gormasz A, Sadoghi P, Mayr W (2020). Wrist movements induce torque and lever force in the scaphoid: an ex vivo study. Journal of Orthopaedic Surgery and Research.

[ref-4] Fennigkoh L, Garg A, Hart B (1999). Mediating effects of wrist reaction torque on grip force production. International Journal of Industrial Ergonomics.

[ref-5] Festen-Schrier VJMM, Amadio PC (2018). The biomechanics of subsynovial connective tissue in health and its role in carpal tunnel syndrome. Journal of Electromyography and Kinesiology.

[ref-6] Filius A, Thoreson AR, Ozasa Y, An K-N, Zhao C, Amadio PC (2017). Delineation of the mechanisms of tendon gliding resistance within the carpal tunnel. Clinical Biomechanics.

[ref-7] Fu T, Cao M, Liu F, Zhu J, Ye D, Feng X, Xu Y, Wang G, Bai Y (2015). Carpal tunnel syndrome assessment with ultrasonography: value of inlet-to-outlet median nerve area ratio in patients versus healthy volunteers. PLOS ONE.

[ref-8] Gabra JN, Domalain M, Li Z-M (2012). Movement of the distal carpal row during narrowing and widening of the carpal arch width. Journal of Biomechanical Engineering.

[ref-9] Gabra JN, Gordon JL, Marquardt TL, Li Z-M (2016). In vivo tissue interaction between the transverse carpal ligament and finger flexor tendons. Medical Engineering & Physics.

[ref-10] Gelberman RH, Hergenroeder PT, Hargens AR, Lundborg GN, Akeson WH (1981). The carpal tunnel syndrome. A study of carpal canal pressures. The Journal of Bone and Joint Surgery.

[ref-11] Genova A, Dix O, Saefan A, Thakur M, Hassan A (2020). Carpal tunnel syndrome: a review of literature. Cureus.

[ref-12] Gupta R, Rowshan K, Chao T, Mozaffar T, Steward O (2004). Chronic nerve compression induces local demyelination and remyelination in a rat model of carpal tunnel syndrome. Experimental Neurology.

[ref-13] Ibrahim I, Khan WS, Goddard N, Smitham P (2012). Carpal tunnel syndrome: a review of the recent literature. The Open Orthopaedics Journal.

[ref-14] Ikeda K, Kaneoka K, Matsunaga N, Ikumi A, Yamazaki M, Yoshii Y (2025). Effects of forearm rotation on wrist flexor and extensor muscle activities. Journal of Orthopaedic Surgery and Research.

[ref-15] Impink BG, Gagnon D, Collinger JL, Boninger ML (2010). Repeatability of ultrasonographic median nerve measures. Muscle & Nerve.

[ref-16] Klauser AS, Halpern EJ, De Zordo T, Feuchtner GM, Arora R, Gruber J, Martinoli C, Löscher WN (2009). Carpal tunnel syndrome assessment with US: value of additional cross-sectional area measurements of the median nerve in patients versus healthy volunteers. Radiology.

[ref-17] Li S, Kociolek AM, Mariano LA, Loh PY (2025). Grip force modulation on median nerve morphology changes. Journal of Orthopaedic Research.

[ref-18] Loh PY, Yeoh WL, Nakashima H, Muraki S (2018). Deformation of the median nerve at different finger postures and wrist angles. PeerJ.

[ref-19] Lopes MM, Lawson W, Scott T, Keir PJ (2011). Tendon and nerve excursion in the carpal tunnel in healthy and CTD wrists. Clinical Biomechanics.

[ref-20] Main EK, Goetz JE, James Rudert M, Goreham-Voss CM, Brown TD (2011). Apparent transverse compressive material properties of the digital flexor tendons and the median nerve in the carpal tunnel. Journal of Biomechanics.

[ref-21] Moriya T, Zhao C, Cha SS, Schmelzer JD, Low PA, An K-N, Amadio PC (2011). Tendon injury produces changes in SSCT and nerve physiology similar to carpal tunnel syndrome in an in vivo rabbit model. Hand.

[ref-22] Ng AJT, Chandrasekaran R, Prakash A, Mogali SR (2022). A systematic review: normative reference values of the median nerve cross-sectional area using ultrasonography in healthy individuals. Scientific Reports.

[ref-23] Oldfield RC (1971). The assessment and analysis of handedness: the Edinburgh inventory. Neuropsychologia.

[ref-24] Plewa K, Potvin JR, Dickey JP (2016). Wrist rotations about one or two axes affect maximum wrist strength. Applied Ergonomics.

[ref-25] Popp WL, Richner L, Lambercy O, Shirota C, Barry A, Gassert R, Kamper DG (2023). Effects of wrist posture and stabilization on precision grip force production and muscle activation patterns. Journal of Neurophysiology.

[ref-26] Presazzi A, Bortolotto C, Zacchino M, Madonia L, Draghi F (2011). Carpal tunnel: normal anatomy, anatomical variants and ultrasound technique. Journal of Ultrasound.

[ref-27] Racine G, Holmes MWR, Kociolek AM (2024). Time-varying changes in median nerve deformation and position in response to quantified pinch and grip forces. Journal of Orthopaedic Research.

[ref-28] Roll SC, Takata SC, Yao B, Kysh L, Mack WJ (2023). Sonographic reference values for median nerve cross-sectional area: a meta-analysis of data from healthy individuals. Journal of Diagnostic Medical Sonography.

[ref-29] Schroen CA, Duey AH, Nasser P, Laudier D, Cagle PJ, Hausman MR (2025). What is the sequence of mechanical and structural failure during stretch injury in the rat median nerve? The neuroclasis classification. Clinical Orthopaedics and Related Research.

[ref-30] Schroen CA, Nasser P, Boecker AH, Cagle PJ, Hausman MR (2026). Second harmonic generation imaging reveals extent of damage in acute nerve stretch injuries in rats. The Journal of Surgical Research.

[ref-31] Seo NJ, Armstrong TJ, Ashton-Miller JA, Chaffin DB (2008). Wrist strength is dependent on simultaneous power grip intensity. Ergonomics.

[ref-32] Turcotte KE, Kociolek AM (2021). Median nerve travel and deformation in the transverse carpal tunnel increases with chuck grip force and deviated wrist position. PeerJ.

[ref-33] Van Doesburg MHM, Henderson J, Mink van der Molen AB, An KN, Amadio PC (2012). Transverse plane tendon and median nerve motion in the carpal tunnel: ultrasound comparison of carpal tunnel syndrome patients and healthy volunteers. PLOS ONE.

[ref-34] Wong AYW, Farias Zuniga A, Keir PJ (2023). Carpal tunnel tendon and sub-synovial connective tissue mechanics are affected by reduced venous return. Clinical Biomechanics.

[ref-35] Xu R, Ren L, Zhang X, Qian Z, Wu J, Liu J, Li Y, Ren L (2024). Non-invasive in vivo study of morphology and mechanical properties of the median nerve. Frontiers in Bioengineering and Biotechnology.

[ref-36] Xu C, Zhou Y, He Z, Liu W, Zou M, Sun Y, Qiu J, Ren Y, Mao G, Wang Y, Xi Q, Chen Y, Zhang B (2022). Difference and ratio of the cross-sectional area of median nerve at the carpal tunnel and the pronator quadratus muscle in diagnosing carpal tunnel syndrome: a cross-sectional study. Annals of Translational Medicine.

[ref-37] Yao B, Roll SC (2022). An ultrasound study of the mobility of the median nerve during composite finger movement in the healthy young wrist. Muscle and Nerve.

[ref-38] Yoshii Y, Villarraga HR, Henderson J, Zhao C, An KN, Amadio PC (2009). Ultrasound assessment of the displacement and deformation of the median nerve in the human carpal tunnel with active finger motion. Journal of Bone and Joint Surgery.

[ref-39] Yoshii Y, Yuine H, Kazuki O, Tung W-L, Ishii T (2015). Measurement of wrist flexion and extension torques in different forearm positions. Biomedical Engineering Online.

[ref-40] Yoshii Y, Zhao C, Amadio PC (2020). Recent advances in ultrasound diagnosis of carpal tunnel syndrome. Diagnostics.

[ref-41] Yoshioka S, Okuda Y, Tamai K, Hirasawa Y, Koda Y (1993). Changes in carpal tunnel shape during wrist joint motion. MRI evaluation of normal volunteers. Journal of Hand Surgery.

